# Towards Efficient Wireless Body Area Network Using Two-Way Relay Cooperation

**DOI:** 10.3390/s18020565

**Published:** 2018-02-13

**Authors:** Maham Waheed, Rizwan Ahmad, Waqas Ahmed, Micheal Drieberg, Muhammad Mahtab Alam

**Affiliations:** 1 School of Electrical Engineering and Computer Science, National University of Sciences and Technology, Islamabad 44000, Pakistan; rizwan.ahmad@seecs.edu.pk; 2 Pakistan Institute of Engineering and Applied Sciences (PIEAS), Islamabad 45650, Pakistan; waqas@pieas.edu.pk; 3 Department of Electrical and Electronics Engineering, Universiti Teknologi PETRONAS, Seri Iskandar 32610, Malaysia; mdrieberg@utp.edu.my; 4 Thomas Johann Seebeck Department of Electronics, Tallinn University of Technology, Tallinn 19086, Estonia; muhammad.alam@ttu.ee

**Keywords:** wireless body area networks, two-way relay cooperation, energy efficiency, quasi-static channel, joint network-channel coding

## Abstract

The fabrication of lightweight, ultra-thin, low power and intelligent body-borne sensors leads to novel advances in wireless body area networks (WBANs). Depending on the placement of the nodes, it is characterized as in/on body WBAN; thus, the channel is largely affected by body posture, clothing, muscle movement, body temperature and climatic conditions. The energy resources are limited and it is not feasible to replace the sensor’s battery frequently. In order to keep the sensor in working condition, the channel resources should be reserved. The lifetime of the sensor is very crucial and it highly depends on transmission among sensor nodes and energy consumption. The reliability and energy efficiency in WBAN applications play a vital role. In this paper, the analytical expressions for energy efficiency (EE) and packet error rate (PER) are formulated for two-way relay cooperative communication. The results depict better reliability and efficiency compared to direct and one-way relay communication. The effective performance range of direct vs. cooperative communication is separated by a threshold distance. Based on EE calculations, an optimal packet size is observed that provides maximum efficiency over a certain link length. A smart and energy efficient system is articulated that utilizes all three communication modes, namely direct, one-way relay and two-way relay, as the direct link performs better for a certain range, but the cooperative communication gives better results for increased distance in terms of EE. The efficacy of the proposed hybrid scheme is also demonstrated over a practical quasi-static channel. Furthermore, link length extension and diversity is achieved by joint network-channel (JNC) coding the cooperative link.

## 1. Introduction

Wireless body area network (WBAN) is comprised of ultra-low-power, self-sustained, intelligent and light weight body-borne sensors, designed to work in/on and around the human body [1]. The technology designed particularly for medical purposes now outstretch to various fields like emergency management, military, sports, entertainment, consumer electronics, and is still carving its way for more [2]. The advancement in the domain has led to the development of intelligent contact lenses, retina prosthesis [3], miniature eyelid stimulator [4], non-invasive glucometer (NGM), implantable insulin pumps, miniature leadless pacemakers [5], implantable cardiac defibrillators (ICDs), wireless capsule endoscopy (WCE), cancer detection and smart wound dressing patches [6]. Apart from medical uses, the progress in the field has resulted in smart fabrics, efficient smart homes and more.

Gartner states that the worldwide wearable devices have generated a revenue of $28.7 billion in 2016, and is expected to grow from 275 million units in 2016 to 477 million units in 2020, generating a revenue of $61.7 billion [7]. The Global Mobile Data Traffic Forecast by Cisco predicts that, by 2020, the number of wearable devices will increase to 601 million globally [8]. According to a survey conducted by Ericsson Consumer Lab (Stockholm, Sweden), 60% of the participants believe that, in next five years, biomedical sensors like smart patches, indigestible pills, and other implantable chips would be commonly used [9].

WBAN consists of small-sized sensors with limited energy resources placed on the body surface or inside the body. The operation of WBAN can be compromised by dynamical channel conditions, arising from changes in temperature, pressure, muscular movement, clothing and varying body postures. Body posture changes result in channel variation and at times may cause deep fades that last longer than usual if a certain body posture sustains [10]. These channel variations can lead to retransmissions due to errors that cause an increase in power consumption and a decrease in the sensor’s life time [11]. The frequent replacement of the sensor’s battery is often undesirable and fosters discomfort to the person.

Therefore, limited energy resources must be used effectively to combat varying channel conditions. To overcome this, channel coding has been proposed to improve the robustness and reliability of communication links. For example, temporal diversity coding (TDC) is used in space and time to enhance the link reliability, and the results have shown a 50% improvement in the throughput [12]. Iterative low-density parity-check (LDPC) decoding is employed to lower the total energy consumption by 20–25% [13]. Raptor codes are used over a frequency-shift keying (FSK) modulated link to make the WBAN more energy efficient with reduced transmitter complexity and lesser implementation cost. The raptor code showed greater efficiency over uncoded FSK and LDPC coded link [14]. The comparison result of raptor code and bose-chaudhuri-hocquenghem (BCH) code in a fading environment showed that the BCH coded data achieves higher energy efficiency for a link length of 10 m or less [15].

Although channel coding is an efficient technique, under deep fades, even the retransmissions are useless and an alternative path is required for the data to be successfully transmitted. In such scenarios, cooperative communication is a viable solution to achieve transmit diversity and improve reliability. The energy efficiency and outage probability for a single relay cooperative communication for an ultra wide band (UWB) based WBAN is discussed in [16]. The results show that in-body sensors can employ cooperation mechanism with on-body sensors to increase energy efficiency and decrease outages. An incremental relay scheme is discussed in [17] with two-stage relay cooperation. The results show an improvement in packet error rate (PER) and energy efficiency (EE) when measured against source-destination hop length. Furthermore, equations are derived for incremental three-stage relay scenario [18] and the simulation results [19] showed an improved PER at the cost of EE. The energy consumption based on various modulation schemes is discussed in [20] for simple and coded WBAN links. Network coding is being used in non-invasive WBANs for improving the reliability of transmission in healthcare units [21]. The paper discusses the signal strength versus bit error rate (BER), but the effect of distance and in-body transmissions is neglected. WBAN is a ubiquitous technology that is evolving at a swift pace. The novel advances and rapid growth in WBAN come with the challenges of robustness, reliability, and efficiency, which needs to be addressed.

The aim of this paper is to analyze various WBAN links for direct and cooperative communication in order to achieve an efficient system. The WBAN systems are not as dense as the conventional wireless sensor networks; therefore, single relay cooperation scenarios are considered. The two-way relay cooperation scenario is introduced, which provides improved results compared to both a direct link and a one-way relay link. The one-way relay scenario is used to provide a diversity path in the case of heavy shadowing or deep fades. The two-way relay scenario is useful when two different parameters are needed for decision-making in a single situation. For example, the cardiological issues are closely related to blood pressure and, in the case of monitoring both electrocardiogram (ECG) and blood pressure, the values are measured by two sensors and coordinated using two-way relay cooperation. A similar case is the coordination between glucometer and insulin pump. Specifically, in this paper, the following contributions are made:

1. The analytical expressions of PER and EE are derived for a two-way relay cooperation scenario. Using realistic transceiver specifications, the optimal packet sizes that provide maximum EE are investigated. Based on this investigation, a hybrid scheme is proposed that allows switching between the three communication modes, namely, direct link, one-way relay cooperation and two-way relay cooperation in terms of EE and optimal packet size. The performance in the case of a quasi-static channel resulting from body posture changes is also shown over the hybrid scheme.
2. A joint network-channel (JNC) coding scheme is applied for various WBAN transmission scenarios using convolutional and BCH codes. The hop length extensions in terms of EE for each of the three communication modes are studied while considering packet overhead.

The existing literature spans EE calculations over direct link [22], incremental relaying with single and two-stage relays [17], and a similar incremental relaying scenario extended to three-stage relay [18,19]. In our work, a two-way relaying scenario is presented particularly for WBAN to reduce the number of transmissions and the number of sensor nodes, which provide better results compared to previously used techniques. A hybrid system, based on these observations, is introduced, which allows switching between the proposed two-way relay and previously defined multi-stage one-way relay and direct link to maximize EE, and also demonstrated over quasi-static channel. To the best of our knowledge, a hybrid scheme and JNC coding have not been proposed before in prior WBAN literature.

The rest of the paper is arranged in the following manner. The system model and channel specifications are described in Section 2. The analysis of system spanning PER and EE expressions is presented in Section 3. The simulation results for PER and EE are shown in Section 4. It also covers the performance of asymmetrical links and optimal packet size for given communication links over specified hop-length. The proposed system that includes all three transmission schemes and can adapt to enhance the energy efficiency of overall system is depicted in Section 5 along with quasi-static channel behavior. Hop-length extension and diversity are achieved by JNC coding in Section 6. Finally, the conclusion is drawn at the end.

## 2. System Model

**Figure 1:**
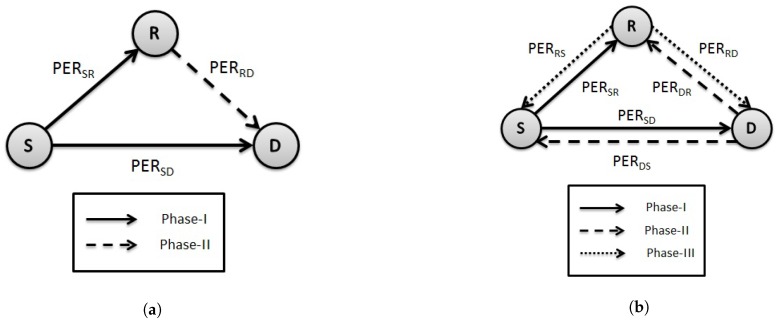
Different communication scenarios using single relay cooperation. (**a**) one-way relay communication; (**b**) two-way relay communication.

A cooperative WBAN scenario is considered, consisting of three sensors mounted in/on the body for various physiological measurements, as shown in Figure 1, with links identified with respective PERs. Similar to the conventional one-way cooperative network, these sensor nodes are called the source *S*, destination *D* and relay *R*. The links between the nodes are considered symmetric and communication is assumed to be half-duplex. Furthermore, all the nodes lie within each other’s transmission range. The main focus is on Tier-1 communication, spanning the area within or around the body up to the radius of about 2 m [23]. The communication architecture, considered as the core of WBAN, is specified by four different channel models, namely, CM1–CM4, based on various in-body and on-body transmission links of WBAN [24].

Two-way relay cooperation occurs between *S* and *D*, in which both nodes exchange their data using *R*. This is different from a conventional one-way cooperative network in which only *S* sends the data to *D* through *R*, as shown in Figure 1a. In the first phase of two-way relay communication, *S* transmits the data to *D*, which is also overheard by *R*. In the second phase, *D* transmits its data to *R*. In the third phase, *R* broadcasts the exclusive-OR (XOR)-ed data of both *S* and *D*. A simple XOR operation is performed at both nodes to get the relevant data. The packet size from both nodes should be of equal length for two-way exchange; otherwise, there would be a need for bit padding. The variable packet size could also be tackled by XOR-ing the bits up to smaller packet length and transmitting the others as it is to avoid overhead. The two-way relay scenario is explained in Figure 1b. The transmission of a packet from *S* to *D* and back to *S* in the case of one-way cooperation requires 4 slots, whereas two-way relay requires only three slots [25]. Thus, providing cooperative diversity while reducing the number of slots.

### Channel Model

In WBAN, multiple transmission links can exist under channel models CM1 to CM4 based on the location of sensor nodes [26]. The instances considered in this work are as follows:

1. In-body communication,
2. On-body line of sight (LOS) communication,
3. On-body non line of sight (NLOS) communication.

In these models, the channel conditions are greatly affected by distant dependent propagation path loss, shadowing, and additive white Gaussian noise (AWGN). The path loss and shadowing are incorporated in channel as follows:

(1) $$P_{L}=PL\left(d\right)+X,$$

where PL(d) is the path loss at a given distance *d* from node and can be defined by Friis formula for free space. *X* is the normally distributed shadowing factor with zero mean and standard deviation sigma. The channel model parameters are defined in Table 1 for in-body communication [24] and on-body communication [27,28]. The parameters include frequency (*f*), reference distance ($d_{0}$), path loss exponent (*n*), shadowing factor (*X*), and reference path loss ($PL(d_{0})$).

## 3. Packet Loss Probability

The packet error rate of a link transmitting packet length of *L* bits from one node to another, along with header of *H* bits, given the bit error probability $P_{b}$ of the link, is expressed as,

(2) $$PER=1-{\left(1-P_{b}\right)}^{L+H}.$$

In two-way relay communication, a packet error occurs in one of the following conditions:

1. Error occurs in both S−D link and S−R link.
2. The direct link (S−D) fails, the relay link (S−R) is error free, data is transmitted from source to relay, but the destination to relay (D−R) link fails.
3. The direct transmission fails i.e., S−D link in error, the relay links from source to relay (S−R) and destination to relay (D−R) are error free, but the broadcast by the relay node fails i.e., either R−D or R−S is in error.

The final equation of PER for the two-way relay link is given below:

(3) $$\begin{array}{c} PER_{TW}=PER_{SD}PER_{SR}+PER_{SD}\left(1-PER_{SR}\right)PER_{DR}\\ +PER_{SD}\left(1-PER_{SR}\right)\left(1-PER_{DR}\right)\left(1-PER_{RS}\right)PER_{RD}. \end{array}$$

The error in any transmission is dependent on the prior transmission or one can say that the successful execution of a phase depends on the previous phase. The broadcast phase in the case of two-way relay scenario is basically protocol dependent and conditioned. Either both links i.e., R−S and R−D fails or any one of them fails to transmit the data. The system would be erroneous in both cases. If both broadcast links fails, no data is received at either end. If one of the links fails, data is collected at one node but again it would not accomplish the two-way cooperation task fully. For a successful accomplishment of two-way relay transmission, both broadcast links should be successful.

### Energy Efficiency

Energy Efficiency is a measure of the useful portion of energy from overall energy consumed during transmission in a link. It is defined as a ratio of the useful fraction of the energy to overall energy consumption and given by Equation (4). The overall energy consumed by data transmission is the sum of energy cost at transmitter, receiver and radio frequency (RF) devices [29]:

(4) $$\eta =\frac{(1-PER)xL}{E_{DATA}+E_{ACK/NACK}},$$

where $x=E_{TX\_elec}+E_{RX\_elec}+\frac{P_{T}}{R}$.

$E_{DATA}$ is the energy consumed in transmission of *L* bits of data, $E_{ACK/NACK}$ is the energy consumed for transmission of acknowledgment packets, $E_{TX\_elec}$ and $E_{RX\_elec}$ are the energy consumed at transmitter and receiver, respectively, during the transmission of a single bit, $P_{T}$ is the transmit power and *R* is the transmission rate.

The energy expenditure on the transmission of data packets over two-way relay is summed up under the following occurrences:

1. The direct link is in the proper working condition and the data is also overheard by the relay.
2. Both direct link and source to relay link fails, but the energy expenditure is similar as in the prior case.
3. The direct link fails, but data is successfully transmitted over relay link. It is then successfully transmitted to target node with probability $PER_{SD}\left(1-PER_{SR}\right)$.
4. The direct link fails, data is successfully transmitted over relay links S−R and D−R, decoded by the relay node *R* and forwarded, but the broadcast link fails.

The final expression of energy expenditure for the two-way relay and transmission of acknowledgment packet containing *A* bits are given in Equations (5) and (6), respectively:

(5) $$\begin{array}{ccc} E_{TW,DATA} & = & [\left(E_{TX\_elec}+2E_{RX\_elec}+\frac{P_{T}}{R}\right)\left(1-PER_{SD}\right)\\ & & +\left(E_{TX\_elec}+2E_{RX\_elec}+\frac{P_{T}}{R}\right)\left(PER_{SD}PER_{SR}\right)\\ & & +\left(2E_{TX\_elec}+3E_{RX\_elec}+2\frac{P_{T}}{R}\right)PER_{SD}\left(1-PER_{SR}\right)\\ & & +\left(3E_{TX\_elec}+5E_{RX\_elec}+3\frac{P_{T}}{R}\right)PER_{SD}\left(1-PER_{SR}\right)\left(1-PER_{DR}\right)\\ & & PER_{RS}PER_{RD}]\left(L+H\right), \end{array}$$

(6) $$\begin{array}{c} E_{TW,ACK/NACK}=[\left(E_{TX\_elec}+2E_{RX\_elec}+\frac{P_{T}}{R}\right)\left(1+PER_{SD}\left(1-PER_{SR}\right)\right]\left(A+H\right). \end{array}$$

Firstly, the energy consumption over error-free direct link is considered. If the direct link is working properly and data is being transmitted to the other node, transmission over relay link is not needed. In the other case, the direct link experiences failure due to shadowing or deep fades and an alternate relay link is required. The second scenario of energy expenditure occurs when both the direct link and the first relay link fail. Another possibility of energy consumption for failed direct link includes the proper working of the first relay link i.e., the data is received at the relay node, after which either it is successfully transmitted to the target node or unsuccessful due to broadcast failure. In two-way relay cooperative scenario, the amount of data sent over the link is doubled as compared to the data sent over the one-way cooperative link.

## 4. Simulation, Performance Evaluation and Optimal Packet Size

The analytical and simulation results for PER and EE are presented against distance for various scenarios, including direct and cooperative communication for a packet size of 500 bits. The analysis is performed on the basis of mathematical expressions formulated in Section 3 using MATLAB (R2015a, MathWorks, Natick, MA, USA). The results are obtained by employing extensive Monte Carlo simulations in MATLAB, in order to substantiate the analytical outcome. A binary phase shift keying (BPSK) modulated bit stream grouped into packets is passed over WBAN link for variable hop-length. The link comprises path loss, log-normal shadowing, and AWGN relative to distance. At the end node, the erroneous packets are differentiated from received packets to evaluate PER. The simulation parameters are listed in Table 2 [17].

**Figure 2:**
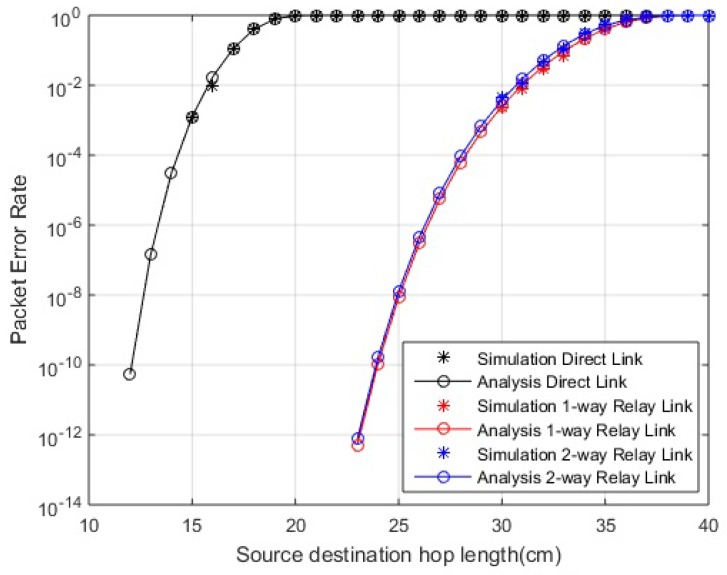
Packet error rate (PER) for in-body communication.

**Figure 3:**
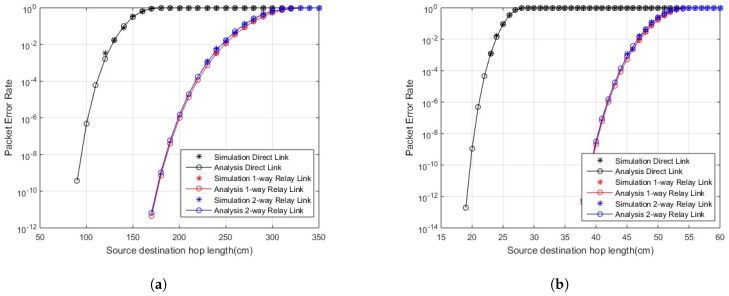
PER for on-body communication; (**a**) PER for on-body line of sight (LOS) communication; (**b**) PER for on-body non line of sight (NLOS) communication.

In Figure 2 and Figure 3, PER for in-body and on-body communication, respectively, is shown over varying link length. The PER is improved in the case of cooperative communication compared to direct link. The one-way and two-way relay curves are equally improved from direct link. Furthermore, in two-way relay communication, the data is shared among both nodes i.e., greater number of transmissions with same PER, so, in two-way relay cooperation scenarios, greater reliability is achieved with lesser errors. A similar result is shown in the case of LOS and NLOS links. The links are reliable for longer hop lengths in the case of LOS links compared to NLOS links.

**Figure 4:**
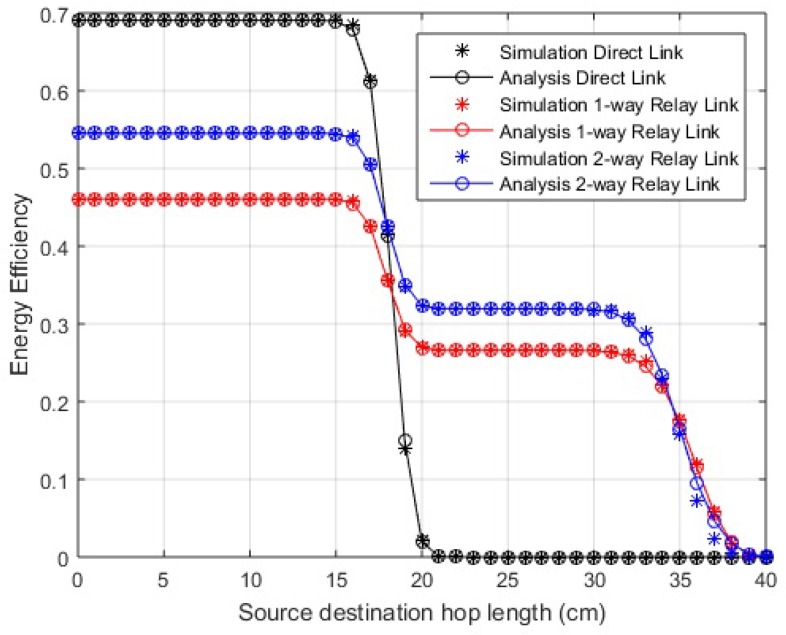
Energy efficiency over distance (in-body communication).

**Figure 5:**
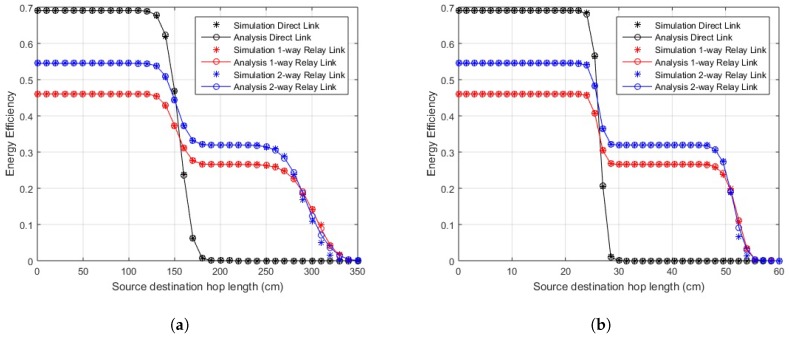
Energy efficiency over distance (on-body communication). (**a**) energy efficiency over distance (on-body LOS communication); (**b**) energy efficiency over distance (on-body NLOS communication).

The EE for transmission over direct and cooperative links is shown in Figure 4 and Figure 5. The energy values of the transceiver are listed in Table 3 [30]. Knowing the energy parameters, the EE is determined as a useful fraction of energy from overall energy consumed. In the case of EE, the optimal policy is of the threshold type. At smaller link distances, we find that EE for the direct link is better than any of the cooperative schemes, but, after a certain threshold level of link length, the cooperative scenarios work better. The EE in the case of two-way relay link is further improved from the one-way relay link as it transmits double the amount of data in a lesser number of transmissions. The communication can be divided into two different regions in terms of EE depending on hop length, the distance where direct communication is better and the distance where better efficiency is obtained through cooperation. A sharp drop in EE values of the direct link is observed when the PER for this link becomes high. The lower EE values of relay links before this threshold value indicate that the losses for relaying are greater for smaller link lengths, but, as the link length increases, they perform efficiently compared to the direct link. It is observed that, approximately around 17 cm, the direct link deteriorates and cooperative links start performing better for in-body communication. Similarly, the direct link’s efficiency decreases above 150 cm and 25 cm for LOS and NLOS on-body communication, respectively. However, still at smaller distances, greater reliability is achieved using cooperation, especially two-way relay cooperation, as we still get better PER than direct links. The simulation results endorse the analytical results.

The existing relaying methods include multiple-stage incremental relaying with the highest efficiency shown by single stage one-way relaying. The proposed two-way relaying outperforms the scheme in terms of energy efficiency, transmission slots and number of sensor nodes.

### 4.1. Performance of Asymmetrical Link

The impact of an asymmetrical link on the performance of the overall system is discussed in this subsection. Two different scenarios are considered in this regard. The placement of relay node i.e., unequal length of S−D link and the R−D link is considered as followed by the unequal amount of data on both links. The simulations are performed in MATLAB using multiple relay positions and by sending unequal data over the links. The results in terms of EE are shown in Table 4 for locating the relay node at distances other than d/2. A comparison of EE shows that the link remains energy efficient over maximum distance when the relay node is placed midway between the source and destination nodes. The percentage decrement in efficient link length, compared with d/2, is visible from Table 4 for all other relay locations. The placement of relay node at the midpoint for achieving maximum EE is also supported in [17] for an incremental one-way relay link.

Table 5 shows the percentage decrement in EE when unequal data is transmitted over both links in two-way relay scenarios. The results show maximum EE when equal data is sent over both links. The percentage decrease in EE, compared to equal data, for different data ratios over the links is elaborated in Table 5.

### 4.2. Optimal Packet Size

WBAN sensors differ in data rate, frequency and payload size depending on the application [31]. The packet size along with other parameters greatly affects the energy efficiency of the system. The consequences of variable packet sizes are elaborated in this subsection.

**Figure 6:**
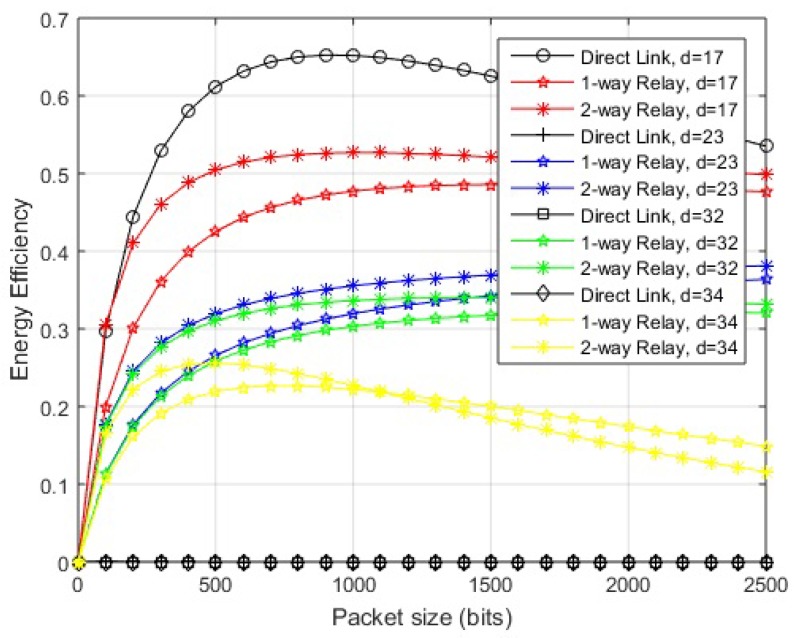
Energy efficiency vs. packet size (in-body communication).

**Figure 7:**
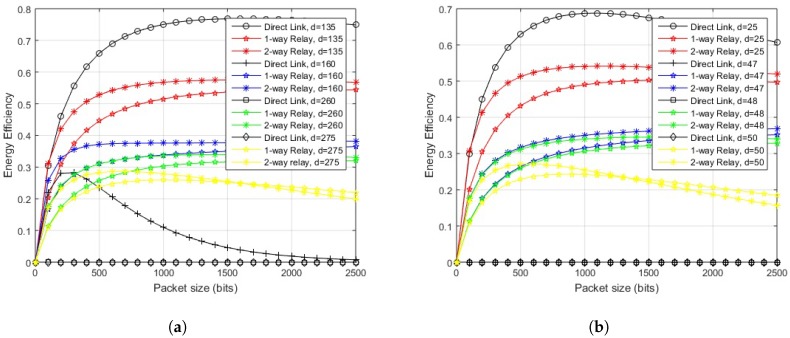
Energy efficiency vs. packet size (on-body communication). (**a**) energy efficiency vs. packet size (on-body LOS communication); (**b**) energy efficiency vs. packet size (on-body NLOS communication).

The optimal packet size can be found by partially differentiating the EE for the given link over packet size. The mathematical expressions obtained could not be shown in closed form easily. Based on the calculations of EE, a suitable packet size for specific link length could be determined by simulations. EE is analyzed for various hop-lengths and multiple packet sizes, with the maximum being 2000 bits as specified by the standard [1], in order to scrutinize the maximum achievable value of efficient energy. The simulation results for different schemes are shown in Figure 6 and Figure 7. A general trend is observed in which greater link lengths reduce the optimal packet size.

In the case of in-body communication, the EE of the direct link remains almost the same for distances less than 17 cm and also greater from cooperative links. At 17 cm, the maximum EE is obtained by transmitting packets with sizes between 500–1000 bits over the direct link. After this distance, a direct link starts to deteriorate in terms of EE and two-way relay link starts performing better. At almost 23 cm, the direct link deteriorates completely. The cooperative links perform well over a range of packet sizes till 32 cm hop length. Afterward, two-way relay performance decreases for larger packets. At approximately 34 cm, the optimal packet size reduces to 500 bits. A further increase of hop length from 35–40 cm results in a decrease of packet size transmitted with greater EE.

Considering on-body LOS communication, the direct link transmits efficiently for hop length of 135 cm with optimal packet size of 1500 bits. The link remains efficient over a packet size of 1000–2000 bits. The cooperative links remain efficient over a range of packet sizes from 160–270 cm. The optimal packet size at 270 cm is 800 bits over a two-way relay link. After this, the two-way relay link deteriorates for larger packets. The one-way relay link performs better for larger packets.

In the case of on-body NLOS communication, the direct communication remains energy efficient for any packet size when source-destination hop length remains less than 20 cm. At 25 cm, we get optimal packet size of 1000 bits approximately on the direct link. Afterwards, cooperative links start to perform better and EE degrades on the direct link even for smaller packets. The cooperative links perform well over a range of packet sizes until the performance starts to degrade for larger packets at about 48 cm, where we get optimal packet size of around 1500–1800 bits. At about 50 cm, the optimal packet size becomes 500 bits and single one-way relay link performs better than a two-way relay link for larger packets. Further increase of hop length from 50–60 cm results in a decrease of packet size transmitted with greater EE.

At this point, the threshold distances for all three communication modes and effective link lengths for various packet sizes are known, so, based on the observations, a smart switching scheme can be formulated. A hybrid distance-based scheme is presented in the next section to maximize the advantage from a specific communication mode. The conditions of the link remain static for a single transmission but vary afterwards resulting in altered source-destination hop-length. The effect of this quasi-static behavior is catered in terms of posture changes. In the case where hop-length exceeds the effective direct link but relay link is not accessible, coding can be used to further improve the effective link length.

## 5. Hybrid Cooperative Scheme for Energy Efficient WBAN

A scheme is presented to adapt a suitable approach, either cooperative or direct, to enhance the energy efficiency of the overall system. The proposed technique takes into account the packet size and source-destination hop-length and determines the type of communication which would maximize the EE of the system. The system comprises of three in/on body sensor nodes, which lay within each other’s transmission range. The transmission is half-duplex and links are symmetric. The pseudocode for the proposed hybrid scheme is described in Algorithm 1. The outcome of the scheme is presented for various hop-lengths over a range of packet sizes, keeping in view the results of PER and EE.

**Figure 8:**
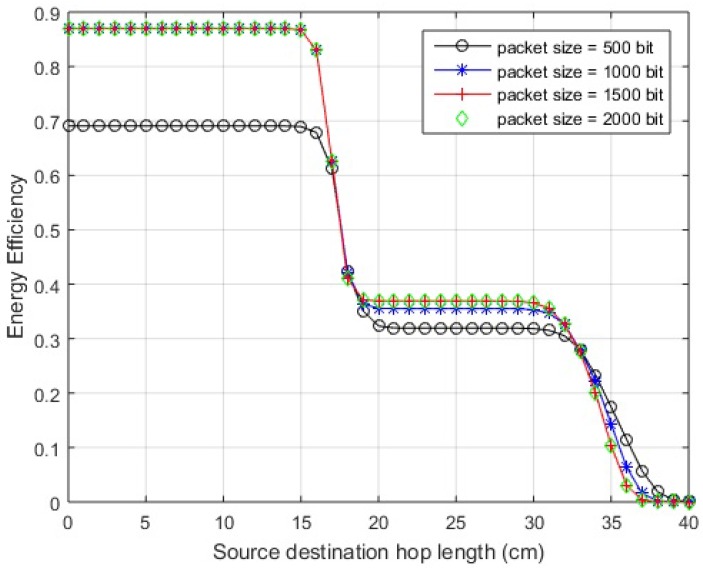
Energy efficiency over distance for proposed scheme (in-body communication).

First, the EE is shown against variable hop length for different packet sizes. The outcome for in-body communication is depicted in Figure 8. The system performs efficiently over the direct link for hop-length of 17 cm or less, for a range of permissible packet sizes. After 17 cm, the direct transmission deteriorates and the link starts to perform better for two-way relay communication until its performance worsens at 35 cm for a packet size of 500 bits, 34 cm for a packet size of 1000 bits, and 33 cm for packet sizes of 1500 and 2000 bits. The one-way relay link performs better afterwards.

**Figure 9:**
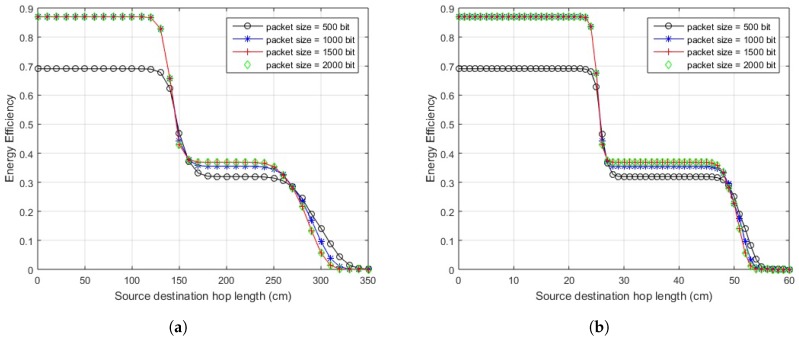
Energy efficiency over distance for proposed scheme (on-body communication). (**a**) energy efficiency over distance for proposed scheme (on-body LOS communication); (**b**) energy efficiency over distance for the proposed scheme (on-body NLOS communication).

Figure 9a illustrates the results of on-body LOS communication. The direct link should be used for a link length of 150 cm or less for 500 bits packet, for all the other packet sizes, the length reduces to 140 cm. The two-way relay link gives better results in terms of EE from 150–290 cm for 500 bits packet. As the size of packet increases to 1000 bits, and 1500 bits, the efficient link length for two-way relay communication reduces to 280 cm and 270 cm, respectively, and remains at 270 cm for larger packets. For a further increase in link lengths, a one-way relay link would outperform both.

In the case of on-body NLOS communication, as depicted in Figure 9b, the direct link should be used for a link length of less than or equal to 25 cm, for a packet size of 500 bits or 1000 bits. For source-destination distance between 26 cm and 51 cm, a two-way relay link would provide better EE. For the link lengths of 51 cm and onwards, a one-way relay link would outperform both. If the packet size is increased to 1500 or 2000 bits, the direct link should be used for a link length of less than or equal to 25 cm, like in the previous case. The link length for two-way relay case reduces from 26 cm to 50 cm, after which one-way relay performs better.

**Algorithm 1** Hybrid cooperative scheme**Require:**
$L=100:100:500,\;d_{in}=0:1:40$ $d_{LOS}=0:10:350,\;d_{NL}=0:1:60$ Where; *L* is payload size and $d_{in}$, $d_{LOS}$ and $d_{LN}$ are the hop-lengths for in-body comm., on-body LOS comm. and on-body NLOS comm. resp. **for**
z=1:length(L)
**do**  **for**
$i=1:length(d_{in})$
**do**   **if**
$d_{in}\left(i\right)\leq 18$
**then**    Use direct link for data transmission    **if**
$\left(18<d_{in}\left(i\right)<36\&L\leq 500\right)$    $∥(18<d_{in}\left(i\right)<35\&500<L<1500)$    $∥(18<d_{in}\left(i\right)<34\&L\geq 1500)$
**then**     Use two-way relay link for data transmission     **if**
$\left(d_{in}\left(i\right)\geq 36\&L\leq 500\right)$     $∥(d_{in}\left(i\right)\geq 35\&500<L<1500)$     $∥(d_{in}\left(i\right)\geq 34\&L\geq 1500)$
**then**      Use one-way relay link for data transmission     **end if**    **end if**   **end if**  **end for**  **for**
$j=1:length(d_{LOS})$
**do**   **if**
$\left(d_{LOS}\left(j\right)\leq 150\&L\leq 500\right)$   $∥(d_{LOS}\left(j\right)\leq 140\&L>500)$
**then**    Use direct link for data transmission    **if**
$\left(150<d_{LOS}\left(j\right)\leq 290\&L\leq 500\right)$    $∥(150<d_{LOS}\left(j\right)\leq 280\&500<L<1500)$    $∥(150<d_{LOS}\left(j\right)\leq 270\&L\geq 1500)$
**then**     Use two-way relay link for data transmission     **if**
$\left(d_{LOS}\left(j\right)>290\&L\leq 500\right)$     $∥(d_{LOS}\left(j\right)>280\&500<L<1500)$     $∥(d_{LOS}\left(j\right)>270\&L\geq 1500)$
**then**      Use one-way relay link for data transmission     **end if**    **end if**   **end if**  **end for**  **for**
$k=1:length(d_{NL})$
**do**   **if**
$d_{NL}\left(k\right)\leq 25$
**then**    Use direct link for data transmission    **if**
$\left(25<d_{NL}\left(k\right)<51\&L<1500\right)$    $∥(25<d_{NL}\left(k\right)<50\&L\geq 1500)$
**then**     Use two-way relay link for data transmission     **if**
$\left(d_{NL}\left(k\right)\geq 51\&L<1500\right)$     $∥(d_{NL}\left(k\right)\geq 50\&L\geq 1500)$
**then**      Use one-way relay link for data transmission     **end if**    **end if**   **end if**  **end for** **end for**

**Figure 10:**
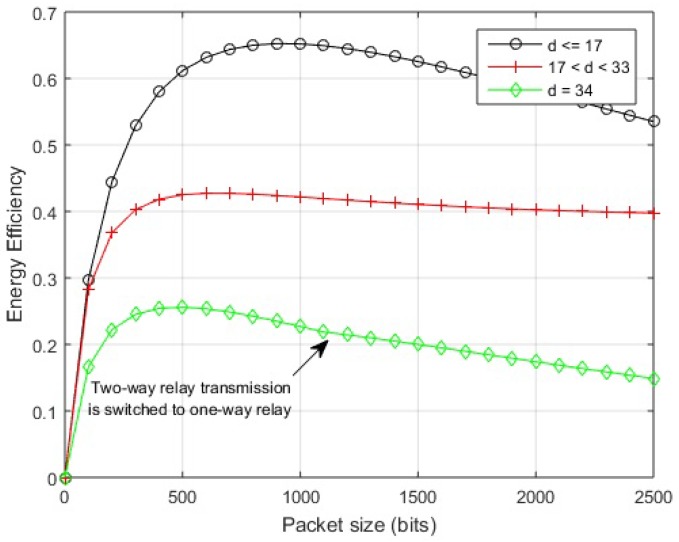
Energy efficiency vs. packet size for proposed scheme (in-body communication).

The impact of variable packet sizes over different link lengths is further elaborated by exhibiting energy efficiency against variable packet sizes for different link lengths. The illustration of in-body communication link in Figure 10 shows that if the link length is 17 cm or less, the direct transmission would provide maximum EE for any permissible packet size. For a link length between 18 and 32 cm, two-way relay transmission gives maximum EE for any number of bits in the packet. After 32 cm, the two-way relay link performs better for 2000 bits packet, which decreases to 1100 bits for 34 cm and so on. Packets with a greater number of bits move more efficiently using a one-way relay link.

**Figure 11:**
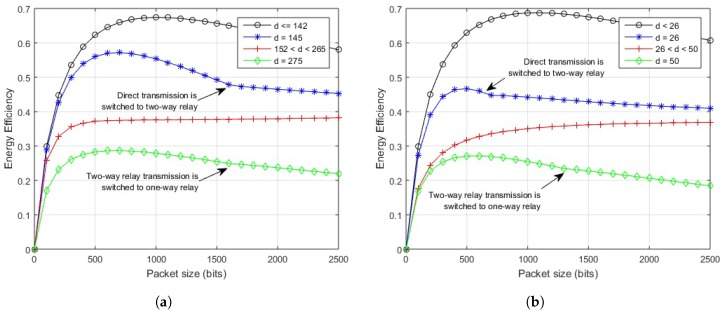
Energy efficiency vs. packet size for the proposed scheme (on-body communication). (**a**) energy efficiency vs. packet size for proposed scheme (on-body LOS communication); (**b**) energy efficiency vs. packet size for proposed scheme (on-body NLOS communication).

The impact of the proposed scheme on in-body LOS communication is clear from Figure 11a. The direct link provides maximum EE for source-destination length of 142 cm and less, for any number of bits in a packet. If the length between source and destination node is 145 cm, a direct link performs better up to a packet size of 1600 bits, after which two-way relay transmission gives maximum efficiency. Increasing the link distance would decrease the packet size for energy efficient direct transmission until it fully lacks behind two-way relay transmission at 153 cm. From 153 to 265 cm, two-way relay link performs well over a range of packet sizes. At about 266 cm, two-way relay communication starts to perform poorly for larger packets. At about 275 cm, it works well up to 1600 bits packet, which further deteriorates with increased link length and one-way relay performs better for larger packets.

The illustration of on-body NLOS link in Figure 11b shows that if the link length is 25 cm or less, the direct transmission would provide maximum EE for any permissible packet size. If the length between source and destination node is 26 cm, a direct link performs better up to a packet size of 600 bits after which two-way relay transmission gives maximum efficiency. For a link length between 26 and 50 cm, two-way relay transmission gives maximum EE for any number of bits in the packet. After 50 cm, the two-way relay link performs better for 1300 bits packet, which decreases to 800 bits for 51 cm and so on, packets with a greater number of bits move more efficiently using a one-way relay link after that.

### Posture Changes

In practical cases, WBAN channels greatly depend on body postures and geometry [32,33]. To study the quasi-static behavior, various body postures are considered over the defined channel model and proposed hybrid scheme. As the described channel uses distance-based path loss and signal-to-noise ratio (SNR), the geometric distribution of channels associated with various postures should be considered to incorporate the effect of the quasi-static channel.

**Figure 12:**
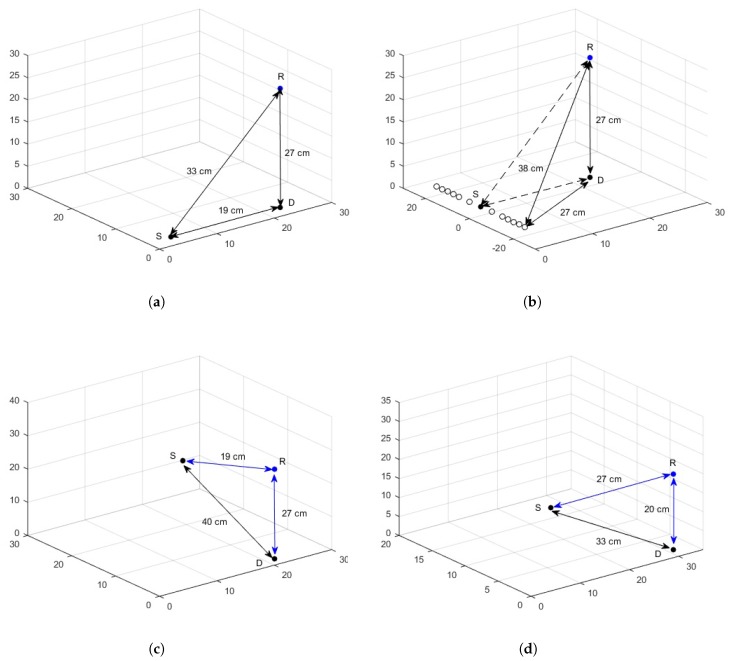
Posture changes. (**a**) standing posture; (**b**) walking posture; (**c**) sleeping posture; (**d**) cycling posture.

A practical scenario is devised to account for posture changes, considering a subject of height 165 cm, trunk radius of 14 cm, arm length of 55 cm, arm radius of 4 cm and arm-body spacing of 2 cm. Three WBAN nodes are deployed on the body with *S* being on the wrist, *R* on the chest and *D* on the waist. The geometric distribution of channel for various postures and its effect on source-destination hop-length is elaborated in Figure 12.

**Figure 13:**
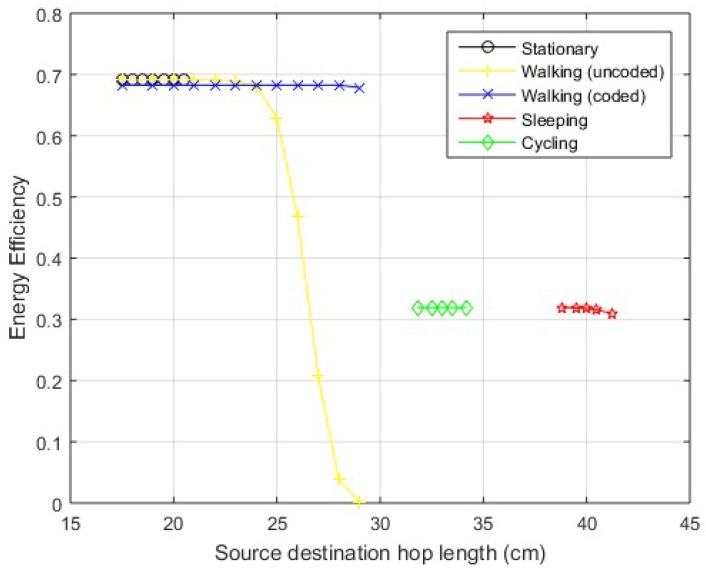
Energy efficiency over posture changes.

*Standing posture:* The graphical interpretation of links between the nodes is shown in Figure 12a. It is clear that the hop-length $d_{SD}$ of direct link (S−D) is smaller than the hop-length $d_{SR}$ of relay link (S−R). Also for the proposed hybrid scheme, $d_{SD}$ lies in the range of efficiently working direct link. Thus, in case of a still standing posture, direct link works and the efficiency over the link can be seen from EE curves of the proposed hybrid scheme in Figure 13 for a link length of 19 cm.

*Walking posture:* In the case of walking, as the arm moves to and fro about the standstill position, the position of *S* node changes resulting in hop-length changes. The calculations show a transition in link length from about 19 cm to 23 cm for a direct link. The direct link ($d_{SD}$) is still smaller than relay link ($d_{SR}$). In addition, for the on-body network, the distance is within the range of efficient direct link and the efficiency over this distance variation can be studied from the EE curve of the hybrid scheme. The geometric interpretation of the posture is shown in Figure 12b, with the solid circle indicating the standing position of *S* node and hollow circles displaying to and fro motion. In the case of greater arm length or larger span of movement, the hop-length might increase further and exceed the effective range of direct communication. It can be noted here that, even after this threshold, the communication does not switch to relay link because $d_{SR}>d_{SD}$. In such cases, JNC is an effective candidate for communicating over the link with extended efficient hop-length. The EE for walking posture is shown in Figure 13 for both uncoded and coded links.

*Sleeping Posture:* A sleeping posture where the hand is placed below the face is depicted in Figure 12c. The posture is considered temporarily stable for longer durations with little movement. The distance between source and destination becomes 40 cm, greater than the range of direct link as proposed by the hybrid scheme for both in-body and on-body networks except LOS. In addition, $d_{SD}>d_{SR}$ so communication would occur over relay link and the efficiency can be seen from proposed hybrid curves with a little deterioration, given the asymmetrical relay link in accordance with Table 4. The EE for the specified posture including the effect of asymmetrical link is shown in Figure 13.

*Cycling Posture:* The body is bent a little forward with the hands held in front in this posture. The graphical representation along with hop-lengths between sensors is shown in Figure 12d and the EE of proposed hybrid scheme is shown in Figure 13. As the distance from direct link exceeds the effective range of the direct link except for the LOS scenario, the transmission occurs over the relay link.

The EE associated with the posture changes described above may show small variations in the already mentioned link lengths as the abdominal displacements occur due to cardiovascular and respiratory cycles. The maximum displacement caused by the mechanical processes is estimated to be 0.2–0.5 mm and 4–12 mm, respectively [34]. The results are shown for on-body NLOS channel, as its distances fall in mid-range and easily elaborated. The variations shown for each posture depicted in Figure 13 includes the described hop-length ±1.2 cm.

In some cases, hop-length exceeds the effective direct link, but relay link is not accessible, as described in walking posture, or the length of relay link exceeds its effective working span of hybrid scheme. Coding can be used over the hybrid scheme in such scenarios. JNC coding is an effective candidate for communicating over such links with an extended efficient hop-length. The effect of JNC coding for various WBAN links is explained in the next section.

## 6. Joint Network-Channel Coding over Cooperative Link

In this section, the performance of joint network-channel coded link is analyzed and simulated. A simple channel coding scheme is used along with network coding to improve the performance of overall system. The existing literature on JNC coding in wireless networks spans multiple access networks [35,36] with 4–5 nodes and two-way relay networks [37,38] using different data combining techniques like superpositioning [39] and XOR-ing [40].

Considering the scenario of WBAN, the number of nodes and power consumption are critical while designing a system and minimum node scenario is a priority, so the said system just considers three nodes and the data is XOR-ed at the relay node. The basic idea behind choosing this scenario is the reduced node deployment and less power consumption for XOR operation. The scheme in which superposition is done on the relay node demands greater energy. The power level is increased at the relay node by superimposing two signals. To bring it back to normal, the power level is scaled at the relay, which, in turn, reduces the power received at receiving nodes. The overall system, hence, loses the performance as the signal received at both of the nodes is less useful.

A similar three nodes scenario is used, where the nodes *S* and *D* want to exchange their data via two-way relay cooperation using relay node *R*. The data to be transmitted among *S* and *D* is indicated by $n_{S}$ and $n_{D}$, respectively.

### 6.1. Joint Network-Channel Coding

In order to transmit the data, it should be channel coded at the sending nodes. The channel coding is performed on both nodes as described,

(7) $$c_{S}=\zeta \left(n_{S}\right),$$

(8) $$c_{D}=\zeta \left(n_{D}\right),$$

where $c_{S}$ indicates the coded data at *S* and $c_{D}$ at *D* while ζ(.) indicates the channel coding function. The data to be shared among the nodes is then BPSK modulated to $x_{S}$ and $x_{D}$ and sent to the relay node in separate slots. The data received at the relay node from the respective nodes is $y_{S}$ and $y_{D}$, where

(9) $$y_{S}=h_{S}x_{S}+z,$$

(10) $$y_{D}=h_{D}x_{D}+z.$$

Here, $h_{S}$ and $h_{D}$ are the channel coefficients of links S−R and D−R, respectively, and *z* accounts for AWGN. The relay node *R* demodulates and decodes the data to get the estimates $\tilde{n_{S}}$ and $\tilde{n_{D}}$. At this point, the relay node has the data of both nodes. Its network encodes the estimates and then the channel encodes them in the following manner:

(11) $$c_{R}=\zeta \left(\tilde{n_{S}}⊕\tilde{n_{D}}\right).$$

If the source coding used is a linear code, then it does not make any difference whether channel coding is done before the network coding or vice versa. Likewise, if the estimated data is channel coded first to $\tilde{c_{S}}$ and $\tilde{c_{D}}$, and network coded later,

(12) $$c_{R}=\tilde{c_{S}}⊕\tilde{c_{D}}=\zeta \left(\tilde{n_{S}}\right)⊕\zeta \left(\tilde{n_{D}}\right)=\zeta \left(\tilde{n_{S}}⊕\tilde{n_{D}}\right).$$

This joint network-channel encoded data is then modulated as $x_{R}$ and broadcasted to both nodes *S* and *D*.

### 6.2. Joint Network-Channel Decoding

In the decoding phase, the data from the relay node *R* has been received by nodes *S* and *D* as a result of the broadcast. The data received at both nodes is given below:

(13) $$y_{RS}=h_{RS}x_{R}+z,$$

(14) $$y_{RD}=h_{RD}x_{R}+z,$$

where $x_{R}$ is the modulated JNC coded data, broadcasted from the relay node, *h* is the channel coefficient and *z* accounts for AWGN. The received data is then demodulated and channel decoded at respective nodes. The estimates $n_{RS}$ and $n_{RD}$ contains the information of both nodes from which original data is extracted in following way:

At node *S*,

(15) $$\tilde{n_{D}}=n_{S}⊕n_{RS},$$

At node *D*,

(16) $$\tilde{n_{S}}=n_{D}⊕n_{RD}.$$

Here, $\tilde{n_{D}}$ specifies the copy of data $n_{D}$ transmitted from node *D*, whereas $\tilde{n_{S}}$ is the copy of data received at node *D* as a result of transmission of $n_{S}$ from node *S*.

### 6.3. Simulation and Performance Evaluation

**Figure 14:**
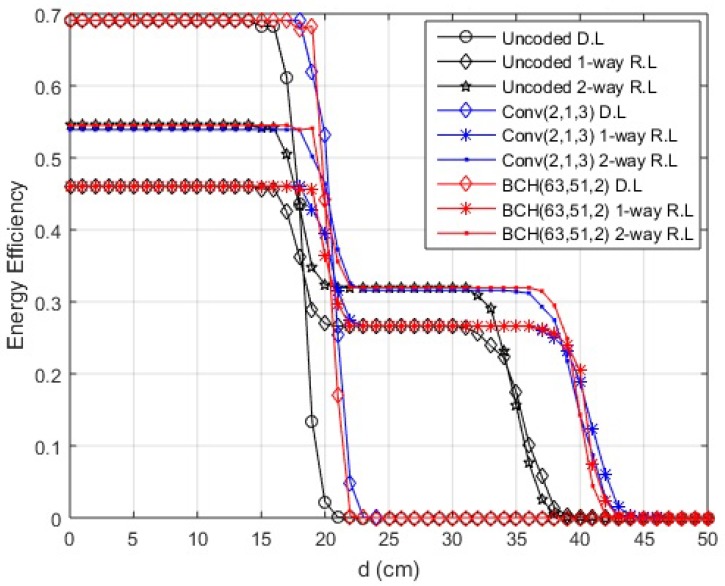
Energy efficiency for joint network channel (JNC) coded data, convolutional R=1/2, BCH t=2 (in-body communication).

**Figure 15:**
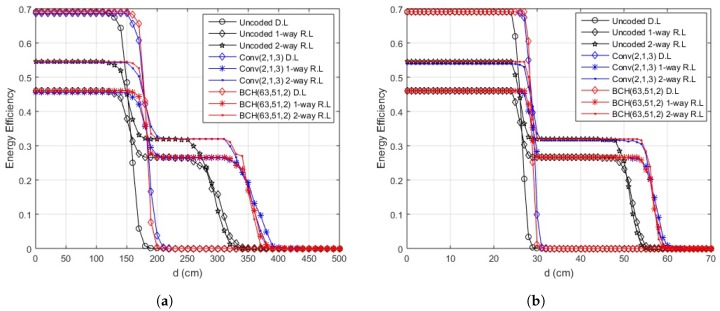
Energy efficiency for JNC coded data, convolutional R=1/2, BCH t=2 (on-body communication). (**a**) energy efficiency for JNC coded data, convolutional R=1/2, BCH t=2 (on-body LOS communication); (**b**) energy efficiency for JNC coded data, convolutional R=1/2, BCH t=2 (on-body NLOS communication).

The range of efficient data transmission is increased using multiple channel coding schemes. As complexity is a major issue in WBAN, simple coding schemes are employed to increase the efficient hop-length and improve reliability. Convolutional codes with code rates of 1/2 and 1/3 and BCH codes with error correction capability of 2 and 4 are studied and analyzed. The simulation results for convolutional codes with R=1/2 and BCH codes with t=2 are shown in Figure 14 for in-body communication and Figure 15 for on-body communication. An extended hop length of 7.5–10% in one-way relay communication and 5–7.5% in two-way relay cooperation for in-body, 8.5–14% for one-way relay communication and 5.5–8.5% for two-way relay cooperation in on-body LOS scenario and 5–7% for one-way relay communication and 3.5–5% for two-way relay cooperation in the case of on-body NLOS is achieved for cooperative communication in the simplest case i.e., convolutional code with code rate 1/2 and BCH codes with error correction capability of 2. Both codes almost result in the same improvement in link length. However, the degradation in case of BCH is more rapid compared to convolutional code.

**Figure 16:**
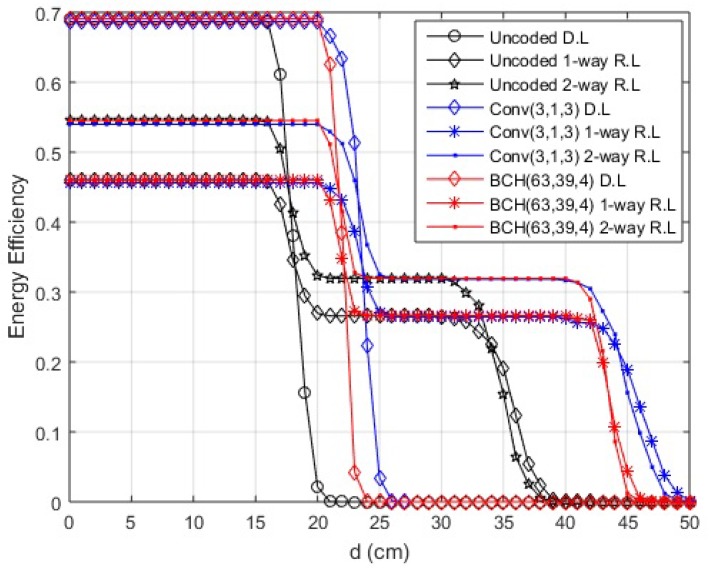
Energy efficiency for JNC coded data, convolutional R=1/3, BCH t=4 (in-body communication).

**Figure 17:**
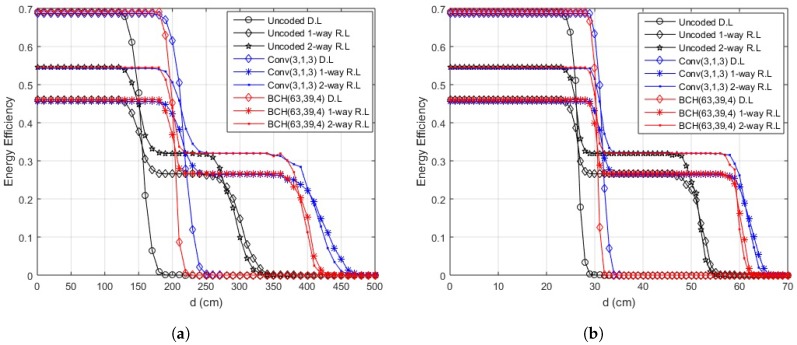
Energy efficiency for JNC coded data, convolutional R=1/3, BCH t=4 (on-body communication). (**a**) energy efficiency for JNC coded data, convolutional R=1/3, BCH t=4 (on-body LOS communication); (**b**) energy efficiency for JNC coded data, convolutional R=1/3, BCH t=4 (on-body NLOS communication).

As the code rate in convolutional codes and error correcting capability in BCH codes increases, the length of link with efficient transmission also increases as depicted by Figure 16 and Figure 17. The maximum extension in hop length is achieved for convolutional codes with a rate of 1/3 but that also means a large overhead i.e., three bits to be transmitted for a single data bit.

The EE of the JNC coded links also depends on the packet size transmitted over the link. The EE results for packet sizes of 500 and 2000 bits show an increase in efficiency for larger data packet i.e., approximately 18–20% improvement in direct link and 11–13% in cooperative link. Similar results are achieved for the EE for BCH coded data for both in-body communication and on-body communication. The improved efficiency is observed at lower link lengths, but smaller packets provide greater hop extension and gradual decay for larger link distances.

#### Asymmetrical Links and Larger Packets

Despite the coding overhead, JNC coding provides an edge in case of asymmetrical links. The communication link gives maximum efficiency if the relay node is placed at equal distance from both source and destination nodes. Change in position of relay node deteriorates the performance of the system and minimizes the efficient length of the link. For example, in the case of in-body cooperation, a 20–35% decrement in efficient transmission length occurs, but if this link is coded, a compensation of 5–10% can be achieved. Similar to Figure 8 and Figure 9, it is observed that larger packet sizes improve the EE at the cost of effective link length. For in-body communication link, approximately 7.5% decrement in effective link length is observed for a packet size of 2000 bits compared to 500 bits. Almost 13–18% improvement in efficient link length can be achieved with JNC coding. This is also true for on-body communication.

A trade-off is observed between efficient link length and overhead. The complexity, encoding and decoding powers will also add to the overhead despite providing efficiency in transmit energy. The choice of the coding scheme would be application dependent, depending on throughput, computational complexity, coding gain and the processing delay it can handle in any given situation to make the overall system reliable and efficient.

## 7. Conclusions

This paper presents the use of two-way relay cooperation along with one-way relay cooperation and direct transmission between WBAN sensors for improved reliability and energy efficiency. Considering Tier-1 communication and practical transmission parameters, the PER and EE are analyzed for all three modes in terms of source destination hop length and relay placement. The results show that an optimal packet size exists in each of the transmission mode that provides maximum EE. Using these results, a hybrid switching scheme including all three modes is devised that provided a gain of approximately 54% in efficient link length compared to direct link for in-body communication, 51.6% for on-body LOS and 50% for on-body NLOS. The system provides cooperative diversity, reduces the error rate, and improves energy efficiency along with a lesser number of implants or wearables. The results show that the hybrid scheme is also useful in quasi-static channel conditions. A JNC scheme is proposed for further improvement in link. It is shown that an extended hop length of 7.5–10% in one-way relay communication and 5–7.5% in two-way relay cooperation for in-body, 8.5–14% for one-way relay communication and 5.5–8.5% for two-way relay cooperation in on-body LOS scenario and 5–7% for one-way relay communication and 3.5–5% for two-way relay cooperation in case of on-body NLOS is achieved for cooperative communication in the simplest case i.e., convolutional code with code rate 1/2 and BCH codes with error correction capability of 2. The results show that selecting moderate code rates for convolution codes and error correcting capability for BCH codes would provide an efficient tradeoff between complexity, overhead and efficiency.

## Figures and Tables

**Table 1:** Channel model parameters.

Parameter	In-Body	On-Body line of sight (LOS)	On-Body non line of sight (NLOS)
f(frequency) (Hz)	402–405 MHz	2.45 GHz	3.1 GHz
$d_{0}\;\left(reference\;distance\right)$ (cm)	5	10	10
n(pathlossexponent)	4.22	3.11	5.9
X(shadowingfactor) (dB)	6.81	6.1	5
$PL\left(d_{0}\right)\;\left(reference\;path\;loss\right)$ (dB)	49.81	35.2	48.4

**Table 2:** Simulation parameters.

Data Packet	500 bits
ACK/NACK	64 bits
Overhead	80 bits
Transmission Power	−10 dBm (in-body)
	−12 dBm (on-body)
Noise Power	−100 dBm
Data Rate	800 kbps (in-body)
	2 Mbps (on-body)
$R/B_{N}$	2 bps/Hz

**Table 3:** Transceiver specifications.

Parameter	In-Body	On-Body
$E_{TX\_elec}$ (nJ/bit)	18.75	11.25
$E_{RX\_elec}$ (nJ/bit)	18.75	11.25

**Table 4:** Relay placement.

Communication Link	Relay Placement	Percentage Decrement		
		In-Body	On-Body LOS	On-Body NLOS
One-way Relay	d/4	32.5%	28.5%	32.1%
	d/3	22.5%	20.0%	23.2%
	2d/3	22.5%	20.0%	21.4%
	3d/4	30.0%	28.5%	30.4%
Two-way Relay	d/4	35.0%	34.3%	33.9%
	d/3	25.0%	25.7%	25.0%
	2d/3	22.5%	20.0%	21.4%
	3d/4	30.0%	28.5%	30.4%

**Table 5:** Unequal data over relay links.

Data Ratio (*S*:*D*)	Percentage Decrement		
	In-Body	On-Body LOS	On-Body NLOS
1:0.8	9.91%	10.09%	10.09%
1:0.75	12.48%	12.48%	12.48%
1:0.5	24.95%	24.95%	24.95%
1:0.25	37.43%	37.43%	37.43%
